# On twelve types of covering-based rough sets

**DOI:** 10.1186/s40064-016-2670-y

**Published:** 2016-07-07

**Authors:** Samira Safari, Mohammad Reza Hooshmandasl

**Affiliations:** Department of Computer Science, Yazd University, Yazd, Iran; The Laboratory of Quantum Information Processing, Yazd University, Yazd, Iran

**Keywords:** Rough sets, Approximation spaces, Covering spaces, Approximation operators

## Abstract

Covering approximation spaces are a generalization of equivalence-based rough set theories. In this paper, we will consider twelve types of covering based approximation operators by combining four types of covering lower approximation operators and three types of covering upper approximation operators. Then, we will study the properties of these new pairs and show they have most of the common properties among existing covering approximation pairs. Finally, the relation between these new pairs is studied.

## Background

Today, we are surrounded by voluminous data which is collected from a wide variety of fields at a great speed. In order to extract useful information from such a great volume of data, which in many cases contains imprecision and uncertainty, the research community has proposed and applied several tools like the theory of rough sets (Pawlak 1982; Pawlak et al. 1988; Pawlak 1991, 1995), and its extensions (Yao 1996, 1998; Skowron and Stepaniuk 1996; Pei and Xu 2004), S-approximation spaces (Hooshmandasl et al. 2014; Shakiba and Hooshmandasl 2015a, b; Shakiba et al. 2016), granular computing (Bargiela and Pedrycz 2003; Lin 2002, 2003) and fuzzy set theory (Zadeh 1965, 1983). Among them, rough set theory is a well-established and popular choice to study information systems.

The theory of rough set was originally proposed by Pawlak (1982) and Pawlak et al. (1988) and has been applied to a wide variety of applications like studying incomplete information systems through coverings (Bonikowski 1994; Bonikowski et al. 1998; Bryniarski 1989; Cattaneo and Ciucci 2005; Kryszkiewicz 1998), granular computing (Lin 2002, 2003; Lin and Liau 2005; lzak and Wasilewski 2007), rule learning (Zhu and Hu 2013; Du et al. 2011) and feature selection (Hu et al. 2008). This theory provides a systematic approach to data analysis through the notion of indiscernibility. The notion of indiscernibility in Pawlak’s original definition is based on equivalence relation, but in many situations in real world, equivalence relations are not applicable. Therefore, this formulation was extended to tolerance (Skowron and Stepaniuk 1996), dominance (Greco et al. 2001), covering (Zakowski 1983; Zhu and Wang 2007), similarity (Slowinski and Vanderpooten 2000), fuzzy (Wu et al. 2003) and arbitrary relations (Yao 1998, 2003). Usually, a concept in rough set theory and its generalizations is approximated by a pair of lower and upper approximations. There are some papers devoted to study the behavior of the lower and upper approximation operators using topology (Zhu 2007; Zhu and Wang 2007).

The covering based rough set theory is a well studied generalized version of rough set theory with important applications such as rule learning (Zhu and Hu 2013; Du et al. 2011) and feature selection (Hu et al. 2008). There exists some types of approximation operator pairs. Zakowski was the first who generalized the Pawlak’s original formulation to covering relations (Zakowski 1983). This model is often called the first type of covering based rough sets. The second type of rough set was proposed by Pomykala along a topological analysis of these approximation spaces (Zhu and Wang 2007), since coverings are a fundamental concept in topological spaces (Zhu 2011). The third type of covering based rough sets were proposed in Tsang et al. (2004) and then studied in Zhu and Wang (2006b) in more details. The fourth type of covering based rough sets were proposed in Zhu and Wang (2012). The fifth pair was introduced in Zhu (2007). There are many approximation pairs for covering rough sets which are studied in Zhang and Luo (2013), Bonikowski (1994), Bonikowski et al. (1998), Bryniarski (1989) and Zhu and Wang (2006a).

In this paper, we take three types of covering based upper approximation operators and then combine them with four types of covering lower based approximation operators, which gives us twelve types of covering approximation operator pairs. Then, we study their properties and compare them to the properties of Pawlaks’s original formulation. Moreover, we study the relation between these new approximation operators.

The organization of this paper is as follows: in “Preliminaries” section, we will review some necessary concepts on rough sets and covering-based approximation spaces. Next, in “Combined types of covering-based rough sets” section, we will introduce twelve new types of covering based approximation pairs. Also, in this section we will investigate basic properties of rough sets for these new types. Then, the results of this section are summarized in two tables. In “Relationships between approximations” section, we will study the relationships between these new types of covering based approximation operators. After giving an illustrative example in “Illustrative example” section, we conclude the paper in “Conclusion and future research directions” section.

## Preliminaries

This section presents a review of some fundamental notions of Pawlak’s rough sets and covering rough sets. We refer to Pawlak (1991) for details.

### Pawlak’s rough set theory

Let *U* be a finite non-empty set and *R* be an equivalence relation over *U*. The equivalence class of *x* with respect to *R* is denoted by $[x]_R$ and is defined as $[x]_R = \{ y \in U | (x,y) \in R \}$. The lower and upper approximations of a set $X \subseteq U$ are defined as

$$\begin{aligned} \underline{R} X = \bigcup \{ [x]_R : [x]_R\subseteq X \} , \end{aligned}$$

and

$$\begin{aligned} \overline{R} X = \bigcup \{ [x]_R : [x]_R \cap X \ne \emptyset \} . \end{aligned}$$

The pair (*U*, *R*) is called a Pawlak approximation space and from the definitions of the approximation sets, the following conclusions have been established.

#### **Proposition 1**

(Pawlak 1991) *LetUbe a finite non-empty set andRan equivalence relation onU*. *Then for any*$X, Y \subseteq U$, *the followings hold:*

(1*L*): $$ \underline{R}(U) =U$$,
(1*H*): $$\overline{R}(U)=U $$,
(2*L*): $$ \underline{R}(\emptyset ) =\emptyset $$,
(2*H*): $$ \overline{R}(\emptyset ) =\emptyset $$,
(3*L*): $$ \underline{R}(X) \subseteq X $$,
(3*H*): $$ X\subseteq \overline{R}(X) $$,
(4*L*): $$ \underline{R}( X \cap Y)=\underline{R}(X)\cap \underline{R}(Y)$$,
(4*H*): $$\overline{R}( X \cup Y)=\overline{R}(X)\cup \overline{R}(Y)$$,
(5*L*): $$ \underline{R}(\underline{R}(X))=\underline{R}(X)$$,
(5*H*): $$\overline{R}(\overline{R}(X))=\overline{R}(X)$$,
(6*L*): $$ X\subseteq Y \Rightarrow \underline{R}(X)\subseteq \underline{R}(Y)$$,
(6*H*): $$ X\subseteq Y \Rightarrow \overline{R}(X)\subseteq \overline{R}(Y)$$,
(7*L*): $$ \underline{R}(-\underline{R}(X))=-\underline{R}(X)$$,
(7*H*): $$ \overline{R}(-\overline{R}(X))=-\overline{R}(X)$$,
(8*LH*): $$\underline{R}(-X)=-\overline{R}(X)$$,
(9*LH*): $$ \underline{R}(X)\subseteq \overline{R}(X)$$,

*where*$$-X = U {\setminus} X$$.

### Covering-based rough set theory

#### **Definition 1**

(*Coverings*) Let *U* be a finite non-empty set and $\mathbf {C}$ be a family of subsets of *U*. Then, $\mathbf {C}$ is called a *covering* of *U* if $K \ne \emptyset$ for every $K \in \mathbf {C}$ and $\bigcup _{K \in \mathbf {C}} K = U$.

For every $x \in U$, the *neighborhood* of *x* induced by $\mathbf {C}$ is defined as $\mathbf {C}_x= \cap \{K \in \mathbf {C} | x \in K \}$. Also, the *minimal description* of *x* with respect to $\mathbf {C}$ is defined as $Md_{\mathbf {C}}(x)=\{K \in \mathbf {C} | x \in K \wedge (\forall S\in \mathbf {C}, x \in S \wedge S\subseteq K \Rightarrow S=K)\}$. The set $CFriends_\mathbf {C}(x) = \cup _{K \in Md_\mathbf {C}(x)}K$ is called the set of *close friends* of *x* with respect to $\mathbf {C}$ (Zhu and Wang 2007). There are plenty of covering based rough approximation operators defined by means of neighborhoods, e.g. Zhu and Wang (2007), Bonikowski et al. (1998) and Zhu and Wang (2012). In the following definition, we will review ten types of them.

#### **Definition 2**

(Bonikowski et al. 1998; Zhu and Wang 2007; Tsang et al. 2004; Zhu and Wang 2012; Zhang and Luo 2013) Let *U* be a finite non-empty set, $\mathbf {C}$ a covering on *U* and $X \subseteq U$. Then,

1. $$\underline{C}_1(X) = \cup \{ K \in \mathbf {C} | K \subseteq X \},$$
  $$\overline{C}_1(X) = \cup \{ CFriends_\mathbf {C}(x) | x \in X \setminus \underline{C}_1(X) \} \cup \underline{C}_1(X).$$
2. $$\underline{C}_2(X) = \underline{C}_1(X),$$
  $$\overline{C}_2(X) = \cup \{ K \in \mathbf {C} | K \cap X\ne \emptyset \}.$$
3. $$\underline{C}_3(X) = \underline{C}_1(X),$$
  $$\overline{C}_3(X) = \cup \{ CFriends_{\mathbf {C}}(x) | x \in X \}.$$
4. $$\underline{C}_4(X) = \underline{C}_1(X),$$
  $$\overline{C}_4(X) = \underline{C}_4(X) \cup \left( \cup \{ K \in \mathbf {C} | K \cap (X \setminus \underline{C}_4(X)) \ne \emptyset \} \right) .$$
5. $$\underline{C}_5(X) = \underline{C}_1(X),$$
  $$\overline{C}_5(X) = \underline{C}_5(X) \cup \left( \cup \{ C_x | x \in X \setminus \underline{C}_5(X) \} \right) .$$
6. $$\underline{C}_6(X) = \{ x \in U | C_x \subseteq X \},$$
  $$\overline{C}_6(X) = \{ x \in U | C_x \cap X \ne \emptyset \}.$$
7. $$\underline{C}_7(X) = \{ x \in U | \forall y \in U \left( x \in C_y \Rightarrow y \in X \right) \},$$
  $$\overline{C}_7(X) = \cup \{ C_x | x \in X \} .$$
8. $$\underline{C}_8(X) = \{ x \in U | \forall K \in \mathbf {C} (x \in K \Rightarrow K \subseteq X) \},$$
  $$\overline{C}_8(X) = \cup \{ K \in \mathbf {C} | K \cap X \ne \emptyset \}.$$
9. $$\underline{C}_9(X) = \{ x \in U | \forall y \in U \setminus X, x \notin CFriend(y) \},$$
  $$\overline{C}_9(X) = \cup \{ CFriend(x) | x \in X \}.$$
10. $$\underline{C}_{10}(X) = \{ x \in U | \forall y \in U, x \in C_y \Rightarrow C_y \subseteq X \},$$
  $$\overline{C}_{10}(X) = \cup \{ C_x | C_x \cap X \ne \emptyset , x \in U \}.$$

In Tables 1 and 2, the basic properties of these covering based approximation operators are summarized.

## Combined types of covering-based rough sets

### **Definition 3**

Let (*U*, *C*) be a covering approximation space. Then, by combining lower approximation operators of types 6, 7, 8, and 10 with upper approximation operators of types 1, 4, and 5, we can define twelve different types of covering based rough set as follows:

1. $$\overline{C}_{61}(X)= \cup \{ CFriends(x) | x \in X - \underline{C_6}(X) \} \cup \underline{C_6}(X),$$
2. $$\overline{C}_{64}(X)=\cup \{K \in C | K \cap (X - \underline{C_6}(X)) \ne \emptyset \} \cup \underline{C_6}(X),$$
3. $$ \overline{C}_{65}(X)= \cup \{C_x | x \in X - \underline{C_6}(X) \} \cup \underline{C_6}(X),$$
4. $$ \overline{C}_{71}(X)= \cup \{ CFriends(x) | x \in X - \underline{C_7}(X) \} \cup \underline{C_7}(X),$$
5. $$ \overline{C}_{74}(X)=\cup \{K \in C | K \cap (X - \underline{C_7}(X)) \ne \emptyset \} \cup \underline{C_7}(X),$$
6. $$ \overline{C}_{75}(X)= \cup \{C_x | x \in X - \underline{C_7}(X) \} \cup \underline{C_7}(X),$$
7. $$ \overline{C}_{81}(X)= \cup \{ CFriends(x) | x \in X - \underline{C_8}(X) \} \cup \underline{C_8}(X),$$
8. $$ \overline{C}_{84}(X)=\cup \{K \in C | K \cap (X - \underline{C_8}(X)) \ne \emptyset \} \cup \underline{C_8}(X) $$
9. $$ \overline{C}_{85}(X)= \cup \{C_x | x \in X - \underline{C_8}(X) \} \cup \underline{C_8}(X),$$
10. $$ \overline{C}_{101}(X)= \cup \{ CFriends(x) | x \in X - \underline{C_{10}}(X) \} \cup \underline{C_{10}}(X),$$
11. $$ \overline{C}_{104}(X)=\cup \{K \in C | K \cap (X - \underline{C_{10}}(X)) \ne \emptyset \} \cup \underline{C_{10}}(X),$$
12. $$ \overline{C}_{105}(X)= \cup \{C_x | x \in X - \underline{C_{10}}(X) \} \cup \underline{C_{10}}(X).$$

Note that the naming convention for $\overline{C}_{xy}(X)$ is as follows:

- *x* represents the type of lower approximation operator in use, e.g. 6, 7, 8, and 10,
- *y* represents the type of upper approximation operator in use, e.g. 1, 4, and 5,

and $$\underline{C}_{xy}(X)=\underline{C}_{x}(X)$$. 

**Table 1:** Properties of lower approximation operations (Zhu and Wang 2012, 2007; Zhang and Luo 2013; Qin et al. 2007)

	$\underline{C}_1$	$\underline{C}_6$	$\underline{C}_7$	$\underline{C}_8$	$\underline{C}_{10}$
1L	$\checkmark$	$\checkmark$	$\checkmark$	$\checkmark$	$\checkmark$
2L	$\checkmark$	$\checkmark$	$\checkmark$	$\checkmark$	$\checkmark$
3L	$\checkmark$	$\checkmark$	$\checkmark$	$\checkmark$	$\checkmark$
4L	–	$\checkmark$	$\checkmark$	$\checkmark$	$\checkmark$
5L	$\checkmark$	$\checkmark$	$\checkmark$	–	–
6L	$\checkmark$	$\checkmark$	$\checkmark$	$\checkmark$	$\checkmark$
7L	–	–	–	–	–

**Table 2:** Properties of upper approximation operations (Zhu and Wang 2012, 2007; Zhang and Luo 2013)

	$\overline{C}_{1}$	$\overline{C}_{2}$	$\overline{C}_{3}$	$\overline{C}_{4}$	$\overline{C}_{5}$	$\overline{C}_{6}$	$\overline{C}_{7}$	$\overline{C}_{8}$	$\overline{C}_{9}$	$\overline{C}_{10}$
1H	$\checkmark$	$\checkmark$	$\checkmark$	$\checkmark$	$\checkmark$	$\checkmark$	$\checkmark$	$\checkmark$	$\checkmark$	$\checkmark$
2H	$\checkmark$	$\checkmark$	$\checkmark$	$\checkmark$	$\checkmark$	$\checkmark$	$\checkmark$	$\checkmark$	$\checkmark$	$\checkmark$
3H	$\checkmark$	$\checkmark$	$\checkmark$	$\checkmark$	$\checkmark$	$\checkmark$	$\checkmark$	$\checkmark$	$\checkmark$	$\checkmark$
4H	–	$\checkmark$	$\checkmark$	–	$\checkmark$	$\checkmark$	$\checkmark$	$\checkmark$	$\checkmark$	$\checkmark$
5H	$\checkmark$	–	–	$\checkmark$	$\checkmark$	$\checkmark$	$\checkmark$	–	–	–
6H	–	$\checkmark$	$\checkmark$	–	$\checkmark$	$\checkmark$	$\checkmark$	$\checkmark$	$\checkmark$	$\checkmark$
7H	–	–	–	–	–	–	–	–	–	–

**Table 3:** Properties of upper approximation operations

	$\overline{C}_{61}$	$\overline{C}_{64}$	$\overline{C}_{65}$	$\overline{C}_{71}$	$\overline{C}_{74}$	$\overline{C}_{75}$	$\overline{C}_{81}$	$\overline{C}_{84}$	$\overline{C}_{85}$	$\overline{C}_{101}$	$\overline{C}_{105}$	
1H	$\checkmark$	$\checkmark$	$\checkmark$	$\checkmark$	$\checkmark$	$\checkmark$	$\checkmark$	$\checkmark$	$\checkmark$	$\checkmark$	$\checkmark$	$\checkmark$
2H	$\checkmark$	$\checkmark$	$\checkmark$	$\checkmark$	$\checkmark$	$\checkmark$	$\checkmark$	$\checkmark$	$\checkmark$	$\checkmark$	$\checkmark$	$\checkmark$
3H	$\checkmark$	$\checkmark$	$\checkmark$	$\checkmark$	$\checkmark$	$\checkmark$	$\checkmark$	$\checkmark$	$\checkmark$	$\checkmark$	$\checkmark$	$\checkmark$
4H	–	–	$\checkmark$	–	–	–	$\checkmark$	$\checkmark$	$\checkmark$	$\checkmark$	–	$\checkmark$
5H	$\checkmark$	$\checkmark$	$\checkmark$	–	–	$\checkmark$	–	–	$\checkmark$	–	–	$\checkmark$
6H	–	–	$\checkmark$	–	–	–	$\checkmark$	$\checkmark$	$\checkmark$	$\checkmark$	-	$\checkmark$
7H	–	–	–	–	–	$\checkmark$	–	–	–	–	–	–

### **Lemma 2**

*Let* (*U*, *C*) *be a covering based approximation space, then for any*$X \subseteq U$, *we have*$\underline{C_6}(\overline{C}_{61}(X))=\overline{C}_{61}(X)$.

### *Proof*

By property (3L) in Proposition (1), $\underline{C_6}(\overline{C}_{61}(X))\subseteq \overline{C}_{61}(X)$. On the other hand, for any $x \in \overline{C}_{61}(X)$, we have $x \in \underline{C_6}(X)$ or $x \in \cup \{ CFriends(x) | x \in X - \underline{C_6}(X) \}$. If $x \in \underline{C_6}(X)$, then $C_x \subseteq X$. By definition of $\overline{C}_{61}(X)$, $X \subseteq \overline{C}_{61}(X)$. Thus $C_x \subseteq \overline{C}_{61}(X)$ and by definition of $\underline{C_6}(X)$, $x \in \underline{C_6}(\overline{C}_{61}(X))$. So $\overline{C}_{61}(X) \subseteq \underline{C_6}(\overline{C}_{61}(X))$.

Also, if $x \in \cup \{ CFriends(x) | x \in X - \underline{C_6}(X) \}$, there exists $x_0 \in X - \underline{C_6}(X)$ such that $x \in CFriends(x_0)$, then $C_x \subseteq CFriends(x_0)$. Since $CFriends(x_0) \subseteq \overline{C}_{61}(X)$, then $C_x \subseteq \overline{C}_{61}(X)$. So $x \in \underline{C_6}(\overline{C}_{61}(X))$. Thus $\overline{C}_{61}(X)\subseteq \underline{C_6}(\overline{C}_{61}(X))$. Therefore, $\underline{C_6}(\overline{C}_{61}(X))=\overline{C}_{61}(X)$. $\square$

### **Proposition 3**

*For*$\overline{C}_{61}(X)$*properties (1H), (2H), (3H) and (5H) do hold.*

### *Proof*

Properties (1H), (2H), and (3H) are directly derivable from definitions. For property (5H), using the property (3H) in Proposition (1), we have $\overline{C}_{61}(X) \subseteq \overline{C}_{61}(\overline{C}_{61}(X))$. On the other hand, for any $x \in \overline{C}_{61}(\overline{C}_{61}(X))$, either $x \in \underline{C_6}(\overline{C}_{61}(X))$ or $x \in \cup \{ CFriends(x) | x \in (\overline{C}_{61}(X) - \underline{C_6}(\overline{C}_{61}(X)) \}$. However, by Lemma (2), $\underline{C_6}(\overline{C}_{61}(X))=\overline{C}_{61}(X)$, which implies that $x \in \overline{C}_{61}(X)$. So $\overline{C}_{61}(\overline{C}_{61}(X))\subseteq \overline{C}_{61}(X)$. $\square$

For $\overline{C}_{61}(X)$ properties (4H), (6H) and (7H) do not hold.

### *Example 1*

Let $U=\{a,b,c,d,e,f,g,h\}$, $X=\{a,b,f,h\}$, $Y=\{a,b,f,h,d\}$ and $C=\{\{f,d,g\},\{a,b,c\},\{a,d,e,f\},\{h\}\}$ is a covering of U. $\overline{C}_{61}(X)=U$, $\overline{C}_{61}(Y)=\{a,b,c,d,f,h\}$ and $\overline{C}_{61}(X \cup Y) =\{a,b,c,d,f,h\}$, thus $\overline{C}_{61}(X \cup Y) \ne \overline{C}_{61}(X) \cup \overline{C}_{61}(Y)$and $X \subseteq Y$, but $\overline{C}_{61}(X)\nsubseteq \overline{C}_{61}(Y)$.

### *Example 2*

Let $U=\{a,b,c,d\}$,$X=\{b,c\}$ and $C=\{\{a,b\},\{b,c,d\},\{c,d\}\}$ is a covering of U. $\overline{C}_{61}(X)=\{b,c,d\}$ then $-\overline{C}_{61}(X)=\{a\}$ and $\overline{C}_{61}(-\overline{C}_{61}(X))=\{a,b\}$. Thus $\overline{C}_{61}(-\overline{C}_{61}(X)) \ne -\overline{C}_{61}(X)$.

### **Lemma 4**

$$ \underline{C_6}(\overline{C}_{64}(X))=\overline{C}_{64}(X)$$.

### *Proof*

By property (3L) in Proposition (1), $\underline{C_6}(\overline{C}_{64}(X))\subseteq \overline{C}_{64}(X)$. On the other hand, for any $x \in \overline{C}_{64}(X)$, we have $x \in \underline{C_6}(X)$ or $x \in \cup \{K \in C | K \cap (X - \underline{C_6}(X)) \ne \emptyset \}$. If $x \in \underline{C_6}(X)$, then $C_x \subseteq X$. By the definitions of $\overline{C}_{64}(X)$, $X \subseteq \overline{C}_{64}(X)$. Thus $C_x \subseteq \overline{C}_{64}(X)$. According to definition $\underline{C_6}(X)$ we have $x \in \underline{C_6}(\overline{C}_{64}(X))$. So $\overline{C}_{64}(X) \subseteq \underline{C_6}(\overline{C}_{64}(X))$.

If $x \in \cup \{K \in C | K \cap (X - \underline{C_6}(X)) \ne \emptyset \}$ there exists $K_i \in C$ such that $K_i \cap (X - \underline{C_6}(X)) \ne \emptyset$ and $x \in K_i$. Since $C_x \subseteq K_i$ and $K_i \subseteq \overline{C}_{64}(X)$, then $C_x \subseteq \overline{C}_{64}(X)$. So $x \in \underline{C_6}(\overline{C}_{64}(X))$, then $\overline{C}_{64}(X)\subseteq \underline{C_6}(\overline{C}_{64}(X))$. Thus, we proved that $\underline{C_6}(\overline{C}_{64}(X))=\overline{C}_{64}(X)$. $\square$

### **Proposition 5**

*For*$\overline{C}_{64}(X)$*properties (1H), (2H), (3H) and (5H) do hold.*

### *Proof*

(1H), (2H), and (3H) are obvious from the definition.

(5H): By property (3H) in Proposition (1), $\overline{C}_{64}(X)\subseteq \overline{C}_{64}(\overline{C}_{64}(X))$.

On the other hand, for any $x \in \overline{C}_{64}(\overline{C}_{64}(X))$, $x \in \underline{C_6}(\overline{C}_{64}(X))$ or $x \in \cup \{K \in C | K \cap (\overline{C}_{64}(X) - \underline{C_6}(\overline{C}_{64}(X)) \ne \emptyset \}$. but By Lemma (4), $\underline{C_6}(\overline{C}_{64}(X)=\overline{C}_{64}(X)$, then $x \in \overline{C}_{64}(X)$. So $\overline{C}_{64}(\overline{C}_{64}(X))\subseteq \overline{C}_{64}(X)$. Therefore, $\overline{C}_{64}(X)= \overline{C}_{64}(\overline{C}_{64}(X))$. $\square$

For $\overline{C}_{64}(X)$ properties (4H), (6H), (7H) do not hold.

### *Example 3*

Let $U=\{a,b,c,d,e,f,g,h\}$, $X=\{a,b,f,h\}$, $Y=\{a,b,f,h,d\}$ and $C=\{\{f,d,g\},\{a,b,c\},\{a,d,e,f\},\{h\}\}$ is a covering of U. $\overline{C}_{64}(X)=U$, $\overline{C}_{64}(Y)=\{a,b,c,d,f,h\}$ and $\overline{C}_{64}(X \cup Y)=\{a,b,c,d,f,h\}$, then $\overline{C}_{64}(X \cup Y) \ne \overline{C}_{64}(X) \cup \overline{C}_{64}(Y)$ and however$X \subseteq Y$, but $\overline{C}_{64}(X)\nsubseteq \overline{C}_{64}(Y)$.

### *Example 4*

Let $U=\{a,b,c,d\}$, $X=\{b,c\}$ and $C=\{\{a,b\},\{b,c,d\},\{c,d\}\}$ is a covering of U. $\overline{C}_{64}(X)=\{b,c,d\}$ and $\overline{C}_{64}(-\overline{C}_{64}(X))=\{a,b\}$. Thus $\overline{C}_{64}(-\overline{C}_{64}(X)) \ne -\overline{C}_{64}(X)$.

### **Theorem 6**

$$ \overline{C}_{65}(X)=\cup \{C_x | x \in X\}$$.

### *Proof*

It is obvious that $\cup \{ C_x | x \in X - \underline{C_6}(X) \} \subseteq \cup \{C_x | x \in X \}$. By property (3L) in Proposition (1), $\underline{C_6}(X) \subseteq X$ and since $X \subseteq \cup \{C_x | x \in X \}$, then $\underline{C_6}(X) \subseteq \cup \{C_x | x \in X \}$. So $\overline{C}_{65}(X)=\cup \{C_x | x \in X - \underline{C_6}(X) \} \cup \underline{C_6}(X) \subseteq \cup \{C_x | x \in X \}$.

On the other hand, it is easy to see $\cup \{C_x | x \in X \} =\cup \{C_x | x \in X-\underline{C_6}(X)\} \cup (\cup \{C_x | x \in \underline{C_6}(X) \}).$ For any $x \in \underline{C_6}(X)$, we have $C_x \subseteq X$. By the definition $\overline{C}_{65}(X)$, $X \subseteq \overline{C}_{65}(X)$, then $C_x \subseteq \overline{C}_{65}(X)$. So $\cup \{C_x | x \in X \}\subseteq \overline{C}_{65}(X)$. We proved that $\overline{C}_{65}(X)=\cup \{C_x | x \in X \}$. $\square$

### **Proposition 7**

*For*$\overline{C}_{65}(X)$*properties (1H), (2H), (3H), (4H), (5H), (6H) do hold.*

### *Proof*

(1H), (2H), and (3H) are obvious from the definition.

(4*H*): By Theorem (6) we have:

$$\begin{aligned} \overline{C}_{65}(X\cup Y)&=\cup \{C_x | x \in (X \cup Y)\} \\ {}&=\cup \{C_x | (x \in X) \cup ( x\in Y)\} \\ {}&=\cup \{C_x | x \in X\} \cup (\cup \{C_x | x \in Y\}) \\ {}&=\overline{C}_{65}(X) \cup \overline{C}_{65}(Y). \end{aligned}$$

(5H): By Theorem (6) we have:

$$\begin{aligned} \overline{C}_{65}(\overline{C}_{65}(X))&=\cup \{C_x | x \in \overline{C}_{65}(X)\} \\ {}&=\cup \{C_x | x \in C_y,y \in X\} \\ {}&=\cup \{C_y | y \in X\} \\ {}&=\overline{C}_{65}(X). \end{aligned}$$

(6H) If $$X \subseteq Y$$ then $$ \overline{C}_{65}(X)=\cup \{C_x | x \in X\}\subseteq \cup \{C_x | x \in Y\}=\overline{C}_{65}(Y)$$. $$\square $$

For $\overline{C}_{65}(X)$ property (7H) does not hold.

### *Example 5*

Let $U=\{a,b,c,d,e\}$, $X=\{a,b,c,d\}$ and $C=\{\{a,b,c\},\{a,b,c,d\},\{d,e\}\}$ is a covering of U. $\overline{C}_{65}(X)=\{a,b,c,d\}$ and $\overline{C}_{65}(-\overline{C}_{65}(X))=\{d,e\}$, thus $\overline{C}_{65}(-\overline{C}_{65}(X)) \ne -\overline{C}_{65}(X)$.

### **Proposition 8**

*For*$\overline{C}_{71}(X)$*properties (1H), (2H) and (3H) do hold.*

### *Proof*

(1H), (2H), and (3H) are obvious from the definition.$\square$

For $\overline{C}_{71}(X)$ properties (4H), (5H), (6H) and (7H) do not hold.

### *Example 6*

Let $U=\{a,b,c,d\}$, $X=\{c,d\}$, $Y=\{d\}$ and $C=\{\{a,b\},\{a,c,d\}\}$ is a covering of U. $\overline{C}_{71}(X)=\{c,d\}$ and $\overline{C}_{71}(Y)=\{a,c,d\}$, thus $\overline{C}_{71}(X \cup Y)=\{c,d\} \ne \overline{C}_{71}(X) \cup \overline{C}_{71}(Y)$and however $X \subseteq Y$, but $\overline{C}_{71}(X)\nsubseteq \overline{C}_{71}(Y)$.

### *Example 7*

In Example (6) let $X=\{d\}$. $\overline{C}_{71}(X)=\{a,c,d\}$ and $\overline{C}_{71}(\overline{C}_{71}(X))=U$ thus $\overline{C}_{71}(\overline{C}_{71}(X))\ne \overline{C}_{71}(X)$.

### *Example 8*

In Example (6) let $X=\{b\}$. $\overline{C}_{71}(X)=\{b\}$ then $-\overline{C}_{71}(X)=\{a,c,d\}$ and $\overline{C}_{71}(-\overline{C}_{71}(X))=U$ thus $\overline{C}_{71}(-\overline{C}_{71}(X))\ne -\overline{C}_{71}(X)$.

### **Proposition 9**

*For*$\overline{C}_{74}(X)$*properties (1H), (2H) and (3H) do hold.*

### *Proof*

(1H), (2H), and (3H) are obvious from the definition.$\square$

For $\overline{C}_{74}(X)$ properties (4H), (6H), (5H) and (7H) do not hold.

### *Example 9*

Let $U=\{a,b,c,d,e\}$ and $C=\{\{a,b,c\},\{a,b,c,d\},\{d,e\}\}$ is a covering of U.if $X=\{a\}$ and $Y=\{a,b,c\}$,then $\overline{C}_{74}(X)=\{a,b,c,d\}$, $\overline{C}_{74}(Y)=\{a,b,c\}$ and $\overline{C}_{74}(X\cup Y)=\{a,b,c\}$. Thus $\overline{C}_{74}(X\cup Y) \ne \overline{C}_{74}(X) \cup \overline{C}_{74}(Y)$. Although $X \subseteq Y$, but $\overline{C}_{74}(X)\nsubseteq \overline{C}_{74}(Y)$.

### *Example 10*

Let $U=\{a,b,c,d\}$, $X=\{b\}$ and $C=\{\{a,b,c\},\{a,d\}\}$ is a covering of U. $\overline{C}_{74}(X)=\{a,b,c\}$ and $\overline{C}_{74}(\overline{C}_{74}(X))=\{a,b,c,d\}$, thus $\overline{C}_{74}(\overline{C}_{74}(X)) \ne \overline{C}_{74}(X)$.

### *Example 11*

In Example (10) let $X=\{d\}$. $\overline{C}_{74}(X)=\{d\}$ and $\overline{C}_{74}(-\overline{C}_{74}(X))=\{a,b,c,d\}$, thus $\overline{C}_{74}(-\overline{C}_{74}(X)) \ne -\overline{C}_{74}(X)$.

### **Proposition 10**

*For*$\overline{C}_{75}(X)$*properties (1H), (2H), (3H), (5H) and (7H) do hold.*

### *Proof*

(1H), (2H), and (3H) are obvious from the definition. $\square$

For $\overline{C}_{75}(X)$ properties (4H) and (6H) do not hold.

### *Example 12*

Let $U=\{a,b,c,d,e,f\}$, $X=\{b,d\}$,$Y=\{a,b,c,d\}$ and $C=\{\{a,b\},\{a,c,d,e\},\{e,f\}\}$ is a covering of U. $\overline{C}_{75}(X)=\{a,b,c,d,e\}$, $\overline{C}_{75}(Y)=\{a,b,c,d\}$ and $\overline{C}_{75}(X \cup Y)=\{a,b,c,d\}$. Thus $\overline{C}_{75}(X \cup Y) \ne \overline{C}_{75}(X)\cup \overline{C}_{75}(Y)$. However $X \subseteq Y$, but $\overline{C}_{75}(X)\nsubseteq \overline{C}_{75}(Y)$.

### **Theorem 11**

$$ \overline{C}_{81}(X)=\cup \{CFriends(x) | x \in X \} $$.

### *Proof*

It is obvious that $\cup \{ CFriends(x) | x \in X - \underline{C_8}(X) \} \subseteq \cup \{CFriends(x) | x \in X \}$. By property (3L) in Proposition (1), $\underline{C_8}(X) \subseteq X$ and since $X \subseteq \cup \{CFriends(x) | x \in X \}$, then $\underline{C_8}(X) \subseteq \cup \{CFriends(x) | x \in X \}$. So $\overline{C}_{81}(X)=\cup \{ CFriends(x) | x \in X - \underline{C_8}(X) \} \cup \underline{C_8}(X) \subseteq \cup \{CFriends(x) | x \in X \}$. On the other hand, it is easy to see

$$\begin{aligned} \cup \{CFriends(x) | x \in X \} =\cup \{CFriends(x) | x \in X-\underline{C_8}(X)\} \cup (\cup \{CFriends(x) | x \in \underline{C_8}(X) \}). \end{aligned}$$

For any $x \in \underline{C_8}(X)$, we have $\forall K \in C(x \in K \Rightarrow K \subseteq X)$. Thus $CFriends(x) \subseteq X$. By the definition $\overline{C}_{81}(X)$, $X \subseteq \overline{C}_{81}(X)$. Then $CFriends(x) \subseteq \overline{C}_{81}(X)$. So $\cup \{CFriends(x) | x \in X \}\subseteq \overline{C}_{81}(X)$.

We proved that $\overline{C}_{81}(X)=\cup \{CFriends(x) | x \in X \}$. $\square$

### **Proposition 12**

*For*$\overline{C}_{81}(X)$*properties (1H), (2H), (3H), (4H) and (6H) do hold.*

### *Proof*

(4H): by Theorem (11) we have:

$$\begin{aligned} \overline{C}_{81}(X \cup Y)&=\cup \{CFriends(x) | x \in (X \cup Y) \} \\&=\cup \{CFriends(x) | x \in X \} \cup (\cup \{CFriends(x) | x \in Y \}) \\&=\overline{C}_{81}(X) \cup \overline{C}_{81}(Y). \end{aligned}$$

(6H): If $X \subseteq Y$, then by Theorem (11)

$$\begin{aligned} \overline{C}_{81}(X)&= \cup \{CFriends(x) | x \in X \} \\&\subseteq \cup \{CFriends(x) | x \in Y \} \\&=\overline{C}_{81}(Y). \end{aligned}$$

$$\square $$

For $\overline{C}_{81}(X)$ properties (5H) and (7H) do not hold.

### *Example 13*

Let $U=\{a,b,c,d,e\}$, $X=\{a,b\}$ and $C=\{\{a,b\},\{a,c,d\},\{d,e\}\}$ is a covering of U. $\overline{C}_{81}(X)=\{a,b,c,d\}$ and $\overline{C}_{81}(\overline{C}_{81}(X))=\{a,b,c,d,e\}$, thus $\overline{C}_{81}(\overline{C}_{81}(X)) \ne \overline{C}_{81}(X)$.

### *Example 14*

In Example (13) $-\overline{C}_{81}(X)=\{e\}$ and $\overline{C}_{81}(-\overline{C}_{81}(X))=\{d,e\}$, thus $\overline{C}_{81}(-\overline{C}_{81}(X)) \ne -\overline{C}_{81}(X)$.

### **Theorem 13**

$$ \overline{C}_{84}(X)=\cup \{K \in C | K \cap X \ne \emptyset \} $$.

### *Proof*

It is obvious that $\cup \{K \in C | K \cap (X - \underline{C_8}(X)) \ne \emptyset \} \subseteq \cup \{K \in C | K \cap X \ne \emptyset \}$. By property (3L) in Proposition (1), $\underline{C_8}(X) \subseteq X$ and since $X \subseteq \cup \{K \in C | K \cap X \ne \emptyset \}$, then $\underline{C_8}(X) \subseteq \cup \{K \in C | K \cap X \ne \emptyset \}$. So $\overline{C}_{84}(X)=\cup \{K \in C | K \cap (X - \underline{C_8}(X)) \ne \emptyset \} \cup \underline{C_8}(X) \subseteq \cup \{K \in C | K \cap X \ne \emptyset \}$. On the other hand, it is easy to see

$$\begin{aligned} \cup \{K \in C | K \cap X \ne \emptyset \}=\cup \{K \in C | K \cap (X-\underline{C_8}(X))\ne \emptyset \} \cup (\cup \{K \in C | K \cap \underline{C_8}(X) \ne \emptyset \}). \end{aligned}$$

For any $x \in \cup \{K \in C | K \cap \underline{C_8}(X) \ne \emptyset \}$ there exists $K \in C$ such that $K \cap \underline{C_8}(X) \ne \emptyset$ and $x \in K$ and $x \in \underline{C_8}(X)$. Since $K \cap \underline{C_8}(X) \ne \emptyset$ then $K \subseteq X$. By the definition of $\overline{C}_{84}(X)$ we have $X \subseteq \overline{C}_{84}(X)$, then $K \subseteq \overline{C}_{84}(X)$, thus $x \in \overline{C}_{84}(X)$. $x \in \underline{C_8}(X)$ and By property (3L) in Proposition (1), $\underline{C_8}(X) \subseteq X$ and by the definition of $\overline{C}_{84}(X)$, $X \subseteq \overline{C}_{84}(X)$, then $x \in \underline{C_8}(X) \subseteq \overline{C}_{84}(X)$.So $\cup \{K \in C | K \cap X \ne \emptyset \} \subseteq \overline{C}_{84}(X)$.

We proved that $\overline{C}_{84}(X)=\cup \{K \in C | K \cap X \ne \emptyset \}$. $\square$

### **Proposition 14**

*For*$\overline{C}_{84}(X)$*properties (1H), (2H), (3H), (4H) and (6H) do hold.*

### *Proof*

(4H) By Theorem (13)

$$\begin{aligned} \overline{C}_{84}(X \cup Y)&=\cup \{K \in C | K \cap (X \cup Y) \ne \emptyset \} \\ {}&=\cup \{K \in C | (K \cap X) \cup (K \cap Y)\ne \emptyset \} \\ {}&=\cup \{K \in C | K \cap X \ne \emptyset \} \cup (\cup \{K \in C | K \cap Y \ne \emptyset \}) \\ {}&=\overline{C}_{84}(X) \cup \overline{C}_{84}(Y). \end{aligned}$$

(6H) If $X \subseteq Y$, then by Theorem (13) $\overline{C}_{84}(X)=\cup \{K \in C | K \cap X \ne \emptyset \} \subseteq \cup \{K \in C | K \cap Y\ne \emptyset \}=\overline{C}_{84}(Y)$.$\square$

For $\overline{C}_{84}(X)$ properties (5H) and (7H) do not hold.

### *Example 15*

Let $U=\{a,b,c,d\}$, $X=\{a,b,c\}$ and $C=\{\{a,b,c\},\{a,b,c,d\},\{d,e\}\}$ is a covering of U. $\overline{C}_{84}(X)=\{a,b,c,d\}$ and $\overline{C}_{84}(\overline{C}_{84}(X))=\{a,b,c,d,e\}$, thus $\overline{C}_{84}(\overline{C}_{84}(X)) \ne \overline{C}_{84}(X)$.

### *Example 16*

In Example (15) $\overline{C}_{84}(X)=\{a,b,c,d\}$ then $-\overline{C}_{84}(X)=\{e\}$ and $\overline{C}_{84}(-\overline{C}_{84}(X))=\{d,e\}$, thus $\overline{C}_{84}(-\overline{C}_{84}(X)) \ne -\overline{C}_{84}(X)$.

### **Theorem 15**

$$ \overline{C}_{85}(X)=\cup \{C_x | x \in X\}$$.

### *Proof*

It is obvious that $\cup \{C_x | x \in X - \underline{C_8}(X)\} \subseteq \cup \{C_x | x \in X\}$. By Property(3L) in Proposition (1), $\underline{C_8}(X) \subseteq X$ and since $X \subseteq \cup \{C_x | x \in X\}$, then $\underline{C_8}(X) \subseteq \cup \{C_x | x \in X\}$. So $\overline{C}_{85}(X)=\cup \{C_x | x \in X - \underline{C_8}(X) \} \cup \underline{C_8}(X) \subseteq \cup \{C_x | x \in X\}$. On the other hand, it is easy to see

$$\begin{aligned} \cup \{C_x | x \in X\}=\cup \{C_x | x \in X - \underline{C_8}(X) \cup (\cup \{C_x | x \in \underline{C_8}(X))\}. \end{aligned}$$

For any $x \in \underline{C_8}(X)$, we have $\forall K \in C(x \in K \Rightarrow K \subseteq X)$. Thus $C_x \subseteq X$. Since $X \subseteq \overline{C}_{85}(X)$, then $C_x \subseteq \overline{C}_{85}(X)$. Therefore, $\cup \{C_x | x \in X\} \subseteq \overline{C}_{85}(X)$.

We proved that $\overline{C}_{85}(X)=\cup \{C_x | x \in X\}$. $\square$

### **Corollary 16**

$$ \overline{C}_{65}(X)= \overline{C}_{85}(X)$$.

### **Proposition 17**

*For*$\overline{C}_{85}(X)$*properties (1H), (2H), (3H), (4H), (5H) and (6H) do hold.*

### *Proof*

(4H): by Theorem (15) we have:

$$\begin{aligned} \overline{C}_{85}(X\cup Y)&=\cup \{C_x | x \in (X \cup Y)\} \\ {}&=\cup \{C_x | (x \in X) \cup ( x\in Y)\} \\ {}&=\cup \{C_x | x \in X\} \cup (\cup \{C_x | x \in Y\}) \\ {}&=\overline{C}_{85}(X) \cup \overline{C}_{85}(Y). \end{aligned}$$

(5H): by Theorem (15) we have:

$$\begin{aligned} \overline{C}_{85}(\overline{C}_{85}(X))&=\cup \left\{ C_x | x \in \overline{C}_{85}(X)\right\} \\&=\cup \left\{ C_x | x \in C_y,y \in X\right\} \\&=\cup \{C_y | y \in X\} \\ {}&=\overline{C}_{85}(X). \end{aligned}$$

(6H): If $$X \subseteq Y$$ then $$ \overline{C}_{85}(X)=\cup \{C_x | x \in X\}\subseteq \cup \{C_x | x \in Y\}=\overline{C}_{85}(Y)$$. $$\square $$

For $\overline{C}_{85}(X)$ property (7H) does not hold.

### *Example 17*

Let $U=\{a,b,c,d,e\}$,$X=\{a,b\}$ and $C=\{\{a,c\},\{a,b\},\{d,e\}\}$ is a covering of U. $\overline{C}_{85}(X)=\{a,b\}$ and $\overline{C}_{85}(-\overline{C}_{85}(X))=\{a,c,d,e\}$, thus $\overline{C}_{85}(-\overline{C}_{85}(X)) \ne -\overline{C}_{85}(X)$.

### **Theorem 18**

$$ \overline{C}_{101}(X)=\cup \{CFriends(x) | x \in X \}$$.

### *Proof*

It is obvious that $\cup \{ CFriends(x) | x \in X - \underline{C_{10}}(X) \} \subseteq \cup \{CFriends(x) | x \in X \}$. By Property(3L) in Proposition (1), $\underline{C_{10}}(X) \subseteq X$ and since $X \subseteq \cup \{C_x | x \in X\}$, then $\underline{C_{10}}(X) \subseteq \cup \{C_x | x \in X\}$. So $\overline{C}_{101}(X)= \cup \{ CFriends(x) | x \in X - \underline{C_{10}}(X) \} \cup \underline{C_{10}}(X) \subseteq \cup \{CFriends(x) | x \in X \}$.

On the other hand, it is easy to see

$$\begin{aligned} \cup \{CFriends(x) | x \in X \}=\cup \{CFriends(x) | x \in X-\underline{C_{10}}(X) \} \cup (\cup \{CFriends(x) | x \in \underline{C_{10}}(X) \}). \end{aligned}$$

For any $x \in \underline{C_{10}}(X)$, we have $\forall y \in U, x \in C_y \implies C_y \subseteq X$. Thus $CFriends(x) \subseteq X$ and from definition $\overline{C}_{101}(X)$, $X \subseteq \overline{C}_{101}(X)$, then $CFriends(x) \subseteq \overline{C}_{101}(X)$. So $\cup \{CFriends(x) | x \in X \} \subseteq \overline{C}_{101}(X)$. Therefore $\overline{C}_{101}(X)=\cup \{CFriends(x) | x \in X \}$. $\square$

### **Corollary 19**

$$\overline{C}_{81}(X)=\overline{C}_{101}(X)$$.

### **Proposition 20**

*For*$\overline{C}_{101}(X)$*properties (1H), (2H), (3H), (4H) and (6H) do hold.*

### *Proof*

(4H): By Theorem (13) we have:

$$\begin{aligned} \overline{C}_{101}(X \cup Y)&=\cup \{CFriends(x) | x \in (X \cup Y) \} \\ {}&=\cup \{CFriends(x) | x \in X \} \cup (\cup \{CFriends(x) | x \in Y \}) \\ {}&=\overline{C}_{101}(X) \cup \overline{C}_{101}(Y). \end{aligned}$$

(6H) If $$X \subseteq Y$$,then $$ \overline{C}_{101}(X)= \cup \{CFriends(x) | x \in X \} \subseteq \cup \{CFriends(x) | x \in Y \} = \overline{C}_{101}(Y)$$. $$\square $$

For $\overline{C}_{101}(X)$ properties (5H) and (7H) do not hold.

### *Example 18*

Let $U=\{a,b,c,d,e\}$,$X=\{a,b\}$ and $C=\{\{a,b\},\{a,c,d\},\{d,e\}\}$ is a covering of U. $\overline{C}_{101}(X)=\{a,b,c,d\}$ and $\overline{C}_{101}(\overline{C}_{101}(X))=\{a,b,c,d,e\}$, thus $\overline{C}_{101}(\overline{C}_{101}(X)) \ne \overline{C}_{101}(X)$.

### *Example 19*

In Example (18) $\overline{C}_{101}(X)=\{a,b,c,d\}$ and $\overline{C}_{101}(-\overline{C}_{101}(X))=\{d,e\}$, thus $\overline{C}_{101}(-\overline{C}_{101}(X)) \ne -\overline{C}_{101}(X)$.

### **Proposition 21**

*For*$\overline{C}_{104}(X)$*properties (1H), (2H) and (3H) do hold.*

### *Proof*

(1H), (2H) and (3H) are obvious from the definition. $\square$

For $\overline{C}_{104}(X)$ properties (4H), (6H), (5H) and (7H) do not hold.

### *Example 20*

Let $U=\{a,b,c,d,e\}$ and $C=\{\{a,b,c\},\{a,b,c,d\},\{d,e\}\}$ is a covering of U. If $X=\{a\}$ and $Y=\{a,b,c\}$, then $\overline{C}_{104}(X)=\{a,b,c,d\}$, $\overline{C}_{104}(Y)=\{a,b,c\}$ and $\overline{C}_{104}(X\cup Y)=\{a,b,c\}$. Thus $\overline{C}_{104}(X\cup Y) \ne \overline{C}_{104}(X) \cup \overline{C}_{104}(Y)$. Although $X \subseteq Y$ , but $\overline{C}_{104}(X)\nsubseteq \overline{C}_{104}(Y)$.

### *Example 21*

In Example (20) $\overline{C}_{104}(X)=\{a,b,c,d\}$ and $\overline{C}_{104}(\overline{C}_{104}(X))=\{a,b,c,d,e\}$, thus $\overline{C}_{104}(\overline{C}_{104}(X)) \ne \overline{C}_{104}(X)$.

### *Example 22*

In Example (20) $\overline{C}_{104}(X)=\{a,b,c,d\}$ and $\overline{C}_{104}(-\overline{C}_{104}(X))=\{d,e\}$, thus $\overline{C}_{104}(-\overline{C}_{104}(X)) \ne -\overline{C}_{104}(X)$.

### **Theorem 22**

$$ \overline{C}_{105}(X)=\cup \{C_x | x \in X\}$$.

### *Proof*

It is obvious that $\cup \{C_x | x \in X - \underline{C_{10}}(X)\} \subseteq \cup \{C_x | x \in X\}$. By property (3L) in Proposition (1), $\underline{C_{10}}(X) \subseteq X$ and since $X \subseteq \cup \{C_x | x \in X\}$, then $\underline{C_{10}}(X) \subseteq \cup \{C_x | x \in X\}$. So $\overline{C}_{105}(X)=\cup \{C_x | x \in X - \underline{C_{10}}(X) \} \cup \underline{C_{10}}(X) \subseteq \cup \{C_x | x \in X\}$. On the other hand, it is easy to see

$$\begin{aligned} \cup \{C_x | x \in X\}=\cup \{C_x | x \in X - \underline{C_{10}}(X) \cup (\cup \{C_x | x \in \underline{C_{10}}(X) )\}. \end{aligned}$$

For any $x \in \underline{C_{10}}(X)$, we have $\forall y (x \in C_y \Rightarrow C_y \subseteq X)$. Since $x \in C_y$ and $X \subseteq \overline{C}_{105}(X)$, then $C_x \subseteq C_y$ and $C_x \subseteq \overline{C}_{105}(X)$. Thus $\cup \{C_x | x \in X\} \subseteq \overline{C}_{105}(X)$. Therefore, $\overline{C}_{105}(X)=\cup \{C_x | x \in X\}$. $\square$

### **Corollary 23**

$$ \overline{C}_{65}(X)= \overline{C}_{85}(X)=\overline{C}_{105}(X)$$.

### **Proposition 24**

*For*$\overline{C}_{105}(X)$*properties (1H), (2H), (3H), (4H), (5H), (6H) do hold.*

### *Proof*

(4H): By Theorem (22)

$$\begin{aligned} \overline{C}_{105}(X\cup Y)&=\cup \{C_x | x \in (X \cup Y)\}\\ {}&=\cup \{C_x | (x \in X) \cup ( x\in Y)\} \\ {}&=\cup \{C_x | x \in X\} \cup (\cup \{C_x | x \in Y\})\\ {}&=\overline{C}_{105}(X) \cup \overline{C}_{105}(Y). \end{aligned}$$

(5H): $$ \overline{C}_{105}(\overline{C}_{105}(X))=\cup \{C_x | x \in \overline{C}_{105}(X)\}=\cup \{C_x | x \in C_y,y \in X\}=\cup \{C_y | y \in X\}=\overline{C}_{105}(X)$$.

(6H): If $$X \subseteq Y$$ then $$ \overline{C}_{105}(X)=\cup \{C_x | x \in X\}\subseteq \cup \{C_x | x \in Y\}=\overline{C}_{105}(Y)$$. $$\square $$

For $\overline{C}_{105}(X)$ property (7H) does not hold.

### *Example 23*

Let $U=\{a,b,c,d\}$, $X=\{a,b\}$ and $C=\{\{a,b,c\},\{a,d\}\}$ is a covering of U. $\overline{C}_{105}(X)=\{a,b,c\}$ and $\overline{C}_{105}(-\overline{C}_{105}(X))=\{a,d\}$, thus $\overline{C}_{105}(-\overline{C}_{105}(X)) \ne -\overline{C}_{105}(X)$.

The results of this section are summarized in Table 3.

## Relationships between approximations

In this section, we will establish the following relationships between the new combined types of coverings for a covering approximation space (*U*, *C*) and $X\subseteq U$:

$$\begin{aligned}&\overline{C}_{75}(X) \subseteq \overline{C}_{85}(X) =\overline{C}_{65}(X)=\overline{C}_{105}(X) \subseteq \overline{C}_{61}(X) \subseteq \overline{C}_{64}(X) \subseteq \overline{C}_{104}(X) \subseteq \overline{C}_{84}(X)\\&\overline{C}_{75}(X) \subseteq \overline{C}_{85}(X)=\overline{C}_{65}(X)=\overline{C}_{105}(X) \subseteq \overline{C}_{61}(X) \subseteq \overline{C}_{81}(X)=\overline{C}_{101}(X) \subseteq \overline{C}_{104}(X) \subseteq \overline{C}_{84}(X)\\&\overline{C}_{75}(X) \subseteq \overline{C}_{71}(X) \subseteq \overline{C}_{101}(X)=\overline{C}_{81}(X) \subseteq \overline{C}_{104}(X) \subseteq \overline{C}_{84}(X)\\&\overline{C}_{75}(X) \subseteq \overline{C}_{71}(X) \subseteq \overline{C}_{74}(X) \subseteq \overline{C}_{84}(X)\\ \end{aligned}$$

### **Theorem 25**

*LetUbe a finite non-empty set*, *Ca covering onUand*$X \subseteq U$. *Then, we have*

1. $$\overline{C}_{65}(X) \subseteq \overline{C}_{61}(X)$$
2. $$\overline{C}_{61}(X) \subseteq \overline{C}_{64}(X)$$
3. $$\overline{C}_{75}(X) \subseteq \overline{C}_{71}(X)$$
4. $$\overline{C}_{71}(X) \subseteq \overline{C}_{74}(X)$$
5. $$\overline{C}_{85}(X) \subseteq \overline{C}_{81}(X)$$
6. $$\overline{C}_{81}(X) \subseteq \overline{C}_{84}(X)$$
7. $$\overline{C}_{105}(X) \subseteq \overline{C}_{101}(X)$$
8. $$\overline{C}_{101}(X) \subseteq \overline{C}_{104}(X)$$
9. $$\overline{C}_{61}(X) \subseteq \overline{C}_{81}(X)$$
10. $$\overline{C}_{64}(X) \subseteq \overline{C}_{104}(X)$$
11. $$\overline{C}_{71}(X) \subseteq \overline{C}_{101}(X)$$
12. $$\overline{C}_{74}(X) \subseteq \overline{C}_{84}(X)$$
13. $$\overline{C}_{74}(X) \subseteq \overline{C}_{104}(X)$$
14. $$\overline{C}_{75}(X) \subseteq \overline{C}_{85}(X)=\overline{C}_{65}(X)=\overline{C}_{105}(X)$$

### *Proof*

1. By the definitions $\overline{C}_{65}(X)$ and $\overline{C}_{61}(X)$, we need only to prove $\cup \{C_x | x \in X - \underline{C_6}(X) \} \subseteq \cup \{ CFriends(x) | x \in X - \underline{C_6}(X) \}$. Since $C_x =\cap Md(x)\subseteq CFriends(x)$, then $\cup \{C_x | x \in X - \underline{C_6}(X) \} \subseteq \cup \{ CFriends(x) | x \in X - \underline{C_6}(X) \}$.
2. By the definitions $\overline{C}_{61}(X)$ and $\overline{C}_{64}(X)$, we need only to prove $\cup \{ CFriends(x) | x \in X - \underline{C_6}(X) \} \subseteq \cup \{K \in C | K \cap (X - \underline{C_6}(X)) \ne \emptyset \}$. For any $x \in X - \underline{C_6}(X)$, there exists $K \in C$ such that $x \in K$. Then $x \in K \cap (X - \underline{C_6}(X))$. Therefore by the definition *CFriends*(*x*) we have $\cup \{ CFriends(x) | x \in X - \underline{C_6}(X) \} \subseteq \cup \{K \in C | K \cap (X - \underline{C_6}(X)) \ne \emptyset \}$.
3. The proof is similar to part (1).
4. The proof is similar to part (2).
5. By Theorem (11) it is obvious that $\cup \{ CFriends(x) | x \in X - \underline{C_6}(X) \}\subseteq \overline{C}_{81}(X)$. By property (3L) in Proposition (1), $\underline{C_6}(X) \subseteq X$ and by property (3H) in Proposition (1), $X \subseteq \overline{C}_{81}(X)$, then $\underline{C_6}(X) \subseteq \overline{C}_{81}(X)$. So $\overline{C}_{61}(X)=\cup \{ CFriends(x) | x \in X - \underline{C_6}(X) \} \cup \underline{C_6}(X) \subseteq \overline{C}_{81}(X)$.
6. By property (3L) in Proposition (1), $\underline{C_6}(X) \subseteq X$ and by property (3H) in Proposition (1), $X \subseteq \overline{C}_{104}(X)$, then $\underline{C_6}(X) \subseteq \overline{C}_{104}(X)$. By Theorem 8 in Qin et al. (2007), $\underline{C_{10}}(X) \subseteq \underline{C_6}(X)$, Then for any $x \in \cup \{K \in C | K \cap (X - \underline{C_6}(X)) \ne \emptyset \}$ we have $x \in \cup \{K \in C | K \cap (X - \underline{C_{10}}(X)) \ne \emptyset \}$. Therefore, $\overline{C}_{64}(X)= \cup \{K \in C | K \cap (X - \underline{C_6}(X)) \ne \emptyset \} \cup \underline{C_6}(X) \subseteq \overline{C}_{104}(X)$.
7. By Theorem (18) it is obvious that $\cup \{ CFriends(x) | x \in X - \underline{C_7}(X) \}\subseteq \overline{C}_{101}(X)$. By property (3L) in Proposition (1), $\underline{C_7}(X) \subseteq X$ and by property (3H) in Proposition (1), $X \subseteq \overline{C}_{101}(X)$, then $\underline{C_7}(X) \subseteq \overline{C}_{101}(X)$. So $\overline{C}_{71}(X)=\cup \{ CFriends(x) | x \in X - \underline{C_7}(X)\} \cup \underline{C_7}(X) \subseteq \overline{C}_{101}(X)$.
8. By Theorem (13) it is obvious that $\cup \{K \in C | K \cap (X - \underline{C_7}(X)) \ne \emptyset \}\subseteq \overline{C}_{84}(X)$. By property (3L) in Proposition (1), $\underline{C_7}(X) \subseteq X$ and by property (3H) in Proposition (1), $X \subseteq \overline{C}_{84}(X)$, then $\underline{C_7}(X) \subseteq \overline{C}_{84}(X)$. So $\overline{C}_{74}(X)=\cup \{K \in C | K \cap (X - \underline{C_7}(X)) \ne \emptyset \} \cup \underline{C_7}(X) \subseteq \overline{C}_{84}(X)$.
9. By property (3L) in Proposition (1), $\underline{C_7}(X) \subseteq X$ and by property (3H) in Proposition (1), $X \subseteq \overline{C}_{104}(X)$, then $\underline{C_7}(X) \subseteq \overline{C}_{104}(X)$. By Theorem 8 in Qin et al. (2007), $\underline{C_{10}}(X) \subseteq \underline{C_7}(X)$, Then for any $x \in \cup \{K \in C | K \cap (X - \underline{C_7}(X)) \ne \emptyset \}$ we have $x \in \cup \{K \in C | K \cap (X - \underline{C_{10}}(X)) \ne \emptyset \}$. Therefore, $\overline{C}_{74}(X)= \cup \{K \in C | K \cap (X - \underline{C_7}(X)) \ne \emptyset \} \cup \underline{C_7}(X) \subseteq \overline{C}_{104}(X)$.
10. By Theorem (6), it is obvious that $\cup \{C_x | x \in X - \underline{C_7}(X) \}\subseteq \overline{C}_{85}(X)$. By property (3L) in Proposition (1), $\underline{C_7}(X) \subseteq X$ and by property (3H) in Proposition (1), $X \subseteq \overline{C}_{85}(X)$, then $\underline{C_7}(X) \subseteq \overline{C}_{85}(X)$. So $\overline{C}_{75}(X)=\cup \{C_x | x \in X - \underline{C_7}(X) \} \cup \underline{C_7}(X) \subseteq \overline{C}_{85}(X)$.

$$\square $$

### **Corollary 26**

*LetUbe a finite non-empty set*, *Ca covering onUand*$X \subseteq U$. *Then, we have*

1. $$\overline{C}_{65}(X) \subseteq \overline{C}_{61}(X)\subseteq \overline{C}_{64}(X)$$
2. $$\overline{C}_{75}(X) \subseteq \overline{C}_{71}(X)\subseteq \overline{C}_{74}(X)$$
3. $$\overline{C}_{85}(X) \subseteq \overline{C}_{81}(X)\subseteq \overline{C}_{84}(X)$$
4. $$\overline{C}_{105}(X) \subseteq \overline{C}_{101}(X)\subseteq \overline{C}_{104}(X)$$
5. $$\overline{C}_{61}(X) \subseteq \overline{C}_{84}(X)$$
6. $$\overline{C}_{65}(X) \subseteq \overline{C}_{104}(X)$$
7. $$\overline{C}_{65}(X) \subseteq \overline{C}_{81}(X)$$
8. $$\overline{C}_{65}(X) \subseteq \overline{C}_{84}(X)$$
9. $$\overline{C}_{71}(X) \subseteq \overline{C}_{104}(X)$$
10. $$\overline{C}_{75}(X) \subseteq \overline{C}_{81}(X) \subseteq \overline{C}_{84}(X)$$
11. $$\overline{C}_{75}(X) \subseteq \overline{C}_{61}(X) \subseteq \overline{C}_{64}(X)$$
12. $$\overline{C}_{75}(X) \subseteq \overline{C}_{101}(X) \subseteq \overline{C}_{104}(X)$$

### *Proof*

1. It follows from parts (1) and (2) of Theorem (25).
2. It follows from parts (3) and (4) of Theorem (25).
3. It follows from parts (5) and (6) of Theorem (25).
4. It follows from parts (7) and (8) of Theorem (25).
5. It follows from parts (5) and (8) of Theorem (25).
6. It follows from part (10) of Theorem (25) and part (1).
7. It follows from part (9) of Theorem (25) and part (1).
8. It follows from part (6) of Theorem (25) and part (7).
9. It follows from parts (8) and (11) of Theorem (25).
10. It follows from part (14) of Theorem (25) and part (3).
11. It follows from part (14) of Theorem (25) and part (1).
12. It follows from part (14) of Theorem (25) and part (4).

$$\square $$

### **Theorem 27**

$$\overline{C}_{104}(X) \subseteq \overline{C}_{84}(X)$$.

### *Proof*

The proof is similar to part (12) of Theorem (25). $\square$

### **Corollary 28**

$$\overline{C}_{64}(X) \subseteq \overline{C}_{84}(X)$$.

### *Proof*

It follows from part (10) of Theorem (25) and Theorem (27). $\square$

### **Corollary 29**

$$\overline{C}_{71}(X) \subseteq \overline{C}_{84}(X)$$.

### *Proof*

It follows from part (9) of Corollary (26) and Theorem (27). $\square$

### **Proposition 30**

$\overline{C}_{61}(X)$*has no relationship with*$\overline{C}_{71}(X)$*and*$\overline{C}_{74}(X)$.

### *Example 24*

Let $U=\{a,b,c,d,e\}$ and $C=\{\{a\},\{a,b\},\{a,c,d\},\{d,e\}\}$ is a covering of U.

If $X=\{a,c,d\}$ then $\overline{C}_{61}(X)=\{a,c,d\}$ and $\overline{C}_{74}(X)=U$, so $\overline{C}_{61}(X) \subseteq \overline{C}_{74}(X)$ .

If $X=\{b\}$ then $\overline{C}_{61}(X)=\{a,b\}$ and $\overline{C}_{74}(X)=\{b\}$, so $\overline{C}_{74}(X) \subseteq \overline{C}_{61}(X)$.

### *Example 25*

In Example (24) let $X=\{b\}$ , then $\overline{C}_{71}(X)=\{b\}$ and $\overline{C}_{61}(X)=\{a,b\}$, so $\overline{C}_{71}(X) \subseteq \overline{C}_{61}(X)$. But, if $X=\{a,c,d\}$, $\overline{C}_{71}(X)=\{a,c,d,e\}$ and $\overline{C}_{61}(X)=\{a,c,d\}$, so $\overline{C}_{61}(X) \subseteq \overline{C}_{71}(X)$.

### **Proposition 31**

$\overline{C}_{64}(X)$*and has no relationship with*$\overline{C}_{71}(X)$, $\overline{C}_{74}(X)$*and*$\overline{C}_{81}(X)$.

### *Example 26*

Let $U=\{a,b,c,d,e\}$ and $C=\{\{a,b,c\},\{a,b,c,d\},\{d,e\}\}$ is a covering of *U* and $X=\{a,b,c,d\}$, then $\overline{C}_{64}(X)=\{a,b,c,d\}$ and $\overline{C}_{81}(X)=\{a,b,c,d,e\}$, so $\overline{C}_{64}(X) \subseteq \overline{C}_{81}(X)$. But, if $X=\{b,c\}$, $\overline{C}_{64}(X)=\{a,b,c,d\}$ and $\overline{C}_{81}(X)=\{a,b,c\}$. So $\overline{C}_{81}(X) \subseteq \overline{C}_{64}(X)$.

### *Example 27*

Let $U=\{a,b,c,d,e\}$ and $C=\{\{a\},\{a,b\},\{a,c,d\},\{d,e\}\}$ is a covering of *U*. If $X=\{b\}$, then $\overline{C}_{71}(X)=\{b\}$ and $\overline{C}_{64}(X)=\{a,b\}$, so $\overline{C}_{71}(X) \subseteq \overline{C}_{64}(X)$. But, if $X=\{a,c,d\}$, $\overline{C}_{71}(X)=\{a,c,d,e\}$ and $\overline{C}_{64}(X)=\{a,c,d\}$. So $\overline{C}_{64}(X) \subseteq \overline{C}_{71}(X)$.

### *Example 28*

In Example (27) if $X=\{b\}$, then $\overline{C}_{74}(X)=\{b\}$ and $\overline{C}_{64}(X)=\{a,b\}$, so $\overline{C}_{74}(X) \subseteq \overline{C}_{64}(X)$. But,if $X=\{a,c,d\}$,$\overline{C}_{74}(X)=\{a,b,c,d,e\}$ and $\overline{C}_{64}(X)=\{a,c,d\}$. So $\overline{C}_{64}(X) \subseteq \overline{C}_{74}(X)$.

### **Proposition 32**

$\overline{C}_{65}(X)=\overline{C}_{85}(X)=\overline{C}_{105}(X)$*has no relationship with*$\overline{C}_{71}(X)$*and*$\overline{C}_{74}(X)$.

### *Example 29*

Let $U=\{a,b,c,d,e\}$ and $C=\{\{a\},\{a,b\},\{a,c,d\},\{d,e\}\}$ is a covering of U. if $X=\{b\}$, then $\overline{C}_{71}(X)=\{b\}$ and $\overline{C}_{65}(X)=\{a,b\}$, so $\overline{C}_{71}(X) \subseteq \overline{C}_{65}(X)$ but, if $X=\{a,c,d\}$, $\overline{C}_{71}(X)=\{a,c,d,e\}$ and $\overline{C}_{65}(X)=\{a,c,d\}$. So $\overline{C}_{65}(X) \subseteq \overline{C}_{71}(X)$.

### *Example 30*

In Example (29) if $X=\{b\}$, then $\overline{C}_{74}(X)=\{b\}$ and $\overline{C}_{65}(X)=\{a,b\}$, so $\overline{C}_{74}(X) \subseteq \overline{C}_{65}(X)$ but, if $X=\{a,c,d\}$, $\overline{C}_{74}(X)=\{a,b,c,d,e\}$ and $\overline{C}_{65}(X)=\{a,c,d\}$. So $\overline{C}_{65}(X) \subseteq \overline{C}_{74}(X)$.

### **Proposition 33**

$\overline{C}_{81}(X)=\overline{C}_{101}(X)$*have no relationship with*$\overline{C}_{64}(X)$*and*$\overline{C}_{74}(X)$,

### *Example 31*

Let $U=\{a,b,c,d,e\}$ and $X=\{b,c\}$, $C=\{\{a\},\{a,b\},\{a,c,d\},\{d,e\}\}$ is a covering of U. $\overline{C}_{74}(X)=\{b,c\}$ and $\overline{C}_{81}(X)=\{a,b,c,d\}$, so $\overline{C}_{74}(X) \subseteq \overline{C}_{81}(X)$. But, if $X=\{a,c,d\}$, $\overline{C}_{74}(X)=U$ and $\overline{C}_{81}(X)=\{a,c,d,e\}$,so $\overline{C}_{81}(X) \subseteq \overline{C}_{74}(X)$.

## Illustrative example

To illustrate the approximation pairs defined so far, the following example is given.

### *Example 32*

Let $U= \left\{ a,b,c,d,e,f,g,h \right\}$ and *C* a covering defined as

$$\begin{aligned} C = \left\{ \{c,d\}, \{f,g\}, \{a,b,c\}, \{c,d,e\}, \{a,b,g\}, \{b,d,g\}, \{a,b,d\}, \{c,g,h\}, \{b,e,h\}\right\} . \end{aligned}$$

For $X = \{a,b,c,d,e\}$, we have

$$\begin{array}{ll} \underline{C}_6(X) = X,&\quad
\overline{C}_6(X) = X, \\ \overline{C}_{61}(X) = X,&\quad
\overline{C}_{64}(X) = X, \\ \overline{C}_{65}(X) = X,&\quad \\
\underline{C}_7(X) = X,&\quad \overline{C}_7(X) = X, \\
\overline{C}_{71}(X) = X,&\quad \overline{C}_{74}(X) = X, \\
\overline{C}_{75}(X) = X,&\quad \\ \underline{C}_8(X) =
\emptyset ,&\quad \overline{C}_8(X) = \{ a,b,c,d,e,g,h \}, \\
\overline{C}_{81}(X) = \{ a,b,c,d,e,g,h \},&\quad
\overline{C}_{84}(X) = \{ a,b,c,d,e,g,h \}, \\ \overline{C}_{85}(X)
= X,&\quad \\ \underline{C}_{10}(X) = X,&\quad \overline{C}_{10}(X)
= X, \\ \overline{C}_{101}(X) = X,&\quad \overline{C}_{104}(X) = X,
\\ \overline{C}_{105}(X) = X.
\end{array}$$

Note that $X \not \in C$. For $Y = \{c,d,e\}$, we have

$$\begin{array}{ll} \underline{C}_6(Y) = Y,&\quad
\overline{C}_6(Y) = Y, \\ \overline{C}_{61}(Y) = Y,&\quad
\overline{C}_{64}(Y) = Y , \\ \overline{C}_{65}(Y) = Y,&\quad \\
\underline{C}_7(Y) = Y,&\quad \overline{C}_7(Y) = Y, \\
\overline{C}_{71}(Y) = Y,&\quad \overline{C}_{74}(Y) = Y, \\
\overline{C}_{75}(Y) = Y,&\quad \\ \underline{C}_8(Y) =
\emptyset ,&\quad \overline{C}_8(Y) = \{ a,b,c,d,e,g,h \}, \\
\overline{C}_{81}(Y) = \{ a,b,c,d,e,g,h \},&\quad
\overline{C}_{84}(Y) = \{ a,b,c,d,e,g,h \}, \\ \overline{C}_{85}(Y)
= \{ c,d,e \},&\quad \\ \underline{C}_{10}(Y) = Y,&\quad
\overline{C}_{10}(Y) = Y, \\ \overline{C}_{101}(Y) = Y,&\quad
\overline{C}_{104}(Y) = Y, \\ \overline{C}_{105}(Y) =
Y.&\end{array}$$

Note that $$Y \in C$$.

## Conclusion and future research directions

In this paper, we proposed a new approach in developing covering based approximation operators using the existing ones, e.g. combination of approximation operators. We used three types of covering based upper approximation operators and then combine them with four types of covering lower based approximation operators, which gives us twelve types of covering approximation operator pairs. The relationships between these new approximation operators is investigated as well as the properties of Pawlaks’s rough set theory.

Possible future research directions include studying topological properties of these new operators; e.g. under which conditions the lower and upper approximation operators coincide with the interior and closure operations in topological spaces, like Zhu and Wang (2006c) and Zhu and Wang (2007). Moreover, topology is used to count the number of different classes of equivalent covering rough sets, which is shown to be equal to the number of topologies of the universe (Ma 2014). Therefore, it seems feasible to apply the same approach to the covering approximation spaces obtained by these pairs of operators. Finally, studying the relation between the covering approximation spaces obtained by these twelve pairs of approximation operators from a topological point, like Zhu and Wang (2006b) and Zhu (2009), of view is another fruitful direction.
